# Small heterodimer partner negatively regulates C-X-C motif chemokine ligand 2 in hepatocytes during liver inflammation

**DOI:** 10.1038/s41598-018-33660-z

**Published:** 2018-10-15

**Authors:** Jung-Ran Noh, Yong-Hoon Kim, Don-Kyu Kim, Jung Hwan Hwang, Kyoung-Shim Kim, Dong-Hee Choi, Seon-Jin Lee, Hee Gu Lee, Tae Geol Lee, Hong-Lei Weng, Steven Dooley, Hueng-Sik Choi, Chul-Ho Lee

**Affiliations:** 1 Laboratory Animal Resource Center, Korea Research Institute of Bioscience and Biotechnology, 125 Gwahak-ro, Yuseong-gu, Daejeon, 34141 South Korea; 2 University of Science and Technology (UST), Daejeon, 34113 South Korea; 3 National Creative Research Initiatives Center for Nuclear Receptor Signals and Hormone Research Center, School of Biological Sciences and Technology, Chonnam National University, Gwangju, 61186 South Korea; 4 Immunotherapy Convergence Research Center, Korea Research Institute of Bioscience and Biotechnology, Daejeon, Republic of Korea; 5 Center for Nano-Bio Measurement, Korea Research Institute of Standard and Science, 267 Gajeong-ro, Yuseong-gu, Daejeon, 34113 South Korea; 6 Department of Medicine II, Section Molecular Hepatology, Medical Faculty Mannheim, Heidelberg University, Theodor-Kutzer Ufer 1-3, 68167 Mannheim, Germany

## Abstract

Recently, we reported that orphan nuclear receptor small heterodimer partner (SHP) is involved in neutrophil recruitment through the regulation of C-X-C motif chemokine ligand 2 (CXCL2) expression in a concanavalin A (ConA)-induced hepatitis model. In the present study, we examined the mechanisms underlying CXCL2 regulation by SHP and the cell types involved in liver inflammation. To this end, either *Shp* knockout (KO) or wild-type (WT) bone marrow cells were transferred into sublethally-irradiated WT (KO → WT or WT → WT) or *Shp* KO (KO → KO or WT → KO) recipients, followed by intravenous injection of ConA (20–30 mg/kg) 8 weeks later. The KO recipient groups showed higher ConA-induced lethality than the WT recipient groups. Accordingly, plasma alanine aminotransferase (ALT) and aspartate aminotransferase (AST) levels, and inflammatory cytokine expressions were significantly higher in the KO recipients than in the WT recipients regardless of donor genotype. Massively increased hepatocyte death in KO recipients, as determined by H&E and TUNEL staining, was observed after ConA challenge. Bone marrow chimera experiments and *in vitro* chemotaxis assay also showed that SHP-deficient hepatocytes have an enhanced ability to recruit neutrophils to the injured liver. *In vitro* promoter assays showed that SHP is a negative regulator of *Cxcl2* transcription by interfering with c-Jun binding to the AP-1 site within the *Cxcl2* promoter. Collectively, SHP regulates *Cxcl2* transcription in hepatocytes, playing a pivotal role in the recruitment of neutrophils. SHP-targeting strategies may represent alternative approaches to control fulminant hepatitis.

## Introduction

Fulminant hepatitis (FH) is a devastating disease that causes severe, often life-threatening, liver failure. It can cause hepatic encephalopathy, coagulopathy, multi-organ failure, and coma^1,2^. Nevertheless, there are currently no therapeutic options other than intensive care and/or orthotopic liver transplantation^3^. The pathogenesis of FH is not fully understood, but immune cell hyperactivity and excessive production of cytokines are important hallmarks of the condition^4^.

Small heterodimer partner (SHP, NR0B2) is an atypical orphan nuclear receptor (NR) with a unique structure and function that is distinct from conventional NRs^5,6^. SHP functions as a corepressor through the heterodimeric interaction with a wide array of nuclear receptors^7,8^. The ability of SHP to target and repress multiple genes in diverse signaling pathways during various biological processes, including the metabolism of bile salts, glucose, and fatty acids is widely accepted^9–13^. However, the role of SHP in innate immunity and inflammation are not yet fully understood.

Previous reports have shown a negative role of SHP in the regulation of innate immunity. During Toll-like receptor (TLR) engagement, proinflammatory cytokines are produced by a series of intracellular mediators, including interleukin-1 receptor-associated kinase 1 (IRAK1), TNF receptor-associated factor 6 (TRAF6), and NF-κB in macrophages^14,15^. SHP is essential for the regulation of the TLR-mediated transactivation of the canonical NF-κB and Lys63-linked polyubiquitination of TRAF6^15^. More recently, we reported that SHP deficiency exacerbates binge drinking-induced liver injury via modulation of innate immune cells such as natural killer T cell and neutrophils^16^. In connection with the effect of SHP on the recruitment of innate immune cells during pathogenic conditions, we have also observed a high susceptibility to ConA-induced inflammatory liver injury in *Shp* KO mice. This phenomenon was attributed to increased neutrophil infiltration into the injured liver, resulting from the expression of the powerful neutrophil chemoattractant, CXCL2^17^. At the time, we could not identify the underlying mechanism for the regulation of CXCL2 expression by SHP. Therefore, in the present study, we examined the specific mechanisms of CXCL2 regulation by SHP in the liver and identified the cell types involved in the recruitment of neutrophils during liver inflammation.

## Materials and Methods

### Animal studies

Eight-week-old male *Shp* KO mice with a C57BL/6 background and WT C57BL/6 control mice were obtained from the Korea Research Institute of Bioscience and Biotechnology (KRIBB; Daejeon, Korea). Bone marrow (BM) chimeric mice were generated by injecting 3 × 10^6^ BM cells into sub-lethally irradiated recipients (900 rad). Eight weeks later, mice were injected intravenously with 20–30 mg/kg of ConA (Sigma-Aldrich Chemical, St Louis, MO, USA). To determine the efficacy of BM cell reconstitution, genomic DNA was extracted from the peripheral blood of chimeric mice and used for *Shp* genotype analysis (Supplementary Fig. 1a). All animal experiments were approved by the Institutional Animal Care and Use Committee of the Korea Research Institute of Bioscience and Biotechnology and were performed in accordance with the Guide for the Care and Use of Laboratory Animals published by the US National Institutes of Health.

### Blood analysis

Plasma alanine aminotransferase (ALT) and aspartate aminotransferase (AST) levels were determined using an automated blood chemistry analyzer (Hitachi 7150; Hitachi, Tokyo, Japan). Total white blood cells in the peripheral blood of bone marrow chimeric mice were counted using an Auto Hematology Analyzer (BC-5300Vet; Mindray, Shenzhen, China) (Supplementary Fig. 1b).

### Histopathology and immunohistochemistry

Liver samples were fixed in 10% neutral-buffered formalin, embedded in paraffin, cut into 5-μm-thick sections, and stained with H&E. To detect neutrophil infiltration, liver sections were stained with an anti-neutrophil antibody (NIMP-R14, Abcam, Cambridge, MA, USA) and visualized using 3,3′-diaminobenzidine (DAB; Vector Lab, Burlingame, CA, USA).

### TUNEL staining

TUNEL staining was performed using paraffin-embedded tissue sections according to the manufacturer’s instructions (Chemicon International, Temecula, CA, USA). Fragmented DNA was deoxygenated by terminal deoxynucleotidyl transferase. The digoxigenin was labeled with anti-digoxigenin-peroxidase and visualized with DAB.

### Quantitative real-time (q)PCR

Total RNA was isolated from mouse livers and reverse transcribed using the iScript^TM^ cDNA Synthesis kit (Bio-Rad, Hercules, CA, USA). The cDNA was subjected to qPCR using the StepOnePlus^TM^ Real-Time PCR System (Applied Biosystems, Foster City, CA, USA) with AccuPower^®^ 2× Greenstar qPCR Master Mix (Bioneer, Daejeon, Korea) according to the manufacturer’s protocol. Relative gene expression levels were analysed using the 2^−ΔΔCt^ method and normalized against the expression of 18 S rRNA. The PCR primer pair sequences are summarized in Supplementary Table 1.

### Isolation of intrahepatic immune cells

Liver samples were collected from euthanized chimeric mice and were forced gently through a 70-μm cell strainer (BD Falcon, San Jose, CA, USA) using a sterile syringe plunger with addition of ice-cold phosphate-buffered saline (PBS). The preparation was centrifuged at 40 × *g* for 5 min at 4 °C. The supernatant was transferred to a new tube and centrifuged at 430 × *g* for 5 min at 4 °C. The pellet was re-suspended in 40% Percoll (GE Healthcare, Buckinghamshire, UK) in PBS and centrifuged at 1,265 × *g* with the no-brake setting for 30 min at 4 °C. The supernatant was discarded and erythrocyte lysis buffer (BioLegend, San Diego, CA, USA) was added to the pellet containing the intrahepatic immune cells. The cells were washed once with fluorescence-activated cell sorting (FACS) buffer prior to analysis by flow cytometry.

### Isolation of blood immune cells

Peripheral blood were collected from euthanized WT or *Shp* KO mice 3 h after PBS or 25 mg/kg of ConA administration. The blood were re-suspended in ice-cold PBS, and centrifuged at 430 × g for 5 min at 4 °C. The supernatant was discarded and erythrocyte lysis buffer (BioLegend) was added to the pellet containing the blood immune cells. The cells were washed once with FACS buffer prior to analysis by flow cytometry.

### Flow-cytometric analysis of intrahepatic or blood immune cells

Cells were washed and Fc receptors were blocked with unlabeled CD16/32 antibody (clone 93; Biolegend). The cells were washed again and extracellular marker proteins were stained for 30 min at 4 °C with fluorophore-conjugated antibodies against CD45 (clone 30-F11), CD11b (clone M1/70), CD3ε (clone 145-2C11), Ly6G (clone 1A8), Ly6C (clone AL-21), CD4 (clone RM4–5), CD8a (53-6.7; all from BD Pharmingen, San Diego, CA, USA), or CD182 (CXCR2; clone SA044G4; BioLegend). The cells were washed twice and analyzed on a Gallios™ Flow Cytometer (Beckman Coulter, Miami, FL, USA). Data were analyzed using the FlowJo software package (TreeStar, San Carlos, CA, USA).

### Bone marrow-derived neutrophils

Mice were euthanized, and bone marrow was collected from femur, tibia, pelvis, scapula, and humerus. Bone marrow neutrophils were isolated by Percoll density gradient centrifugation and hypotonic lysis of red blood cells as previously described. Neutrophils were suspended in RPMI 1640 supplemented with 5% fetal bovine serum (FBS) and held on ice prior to testing. Neutrophil preparations were routinely >75% pure as determined by a Gallios™ Flow Cytometer (Beckman Coulter) (Supplementary Fig. 2).

### Neutrophil chemotaxis assay

WT and *Shp* KO mice were injected intravenously with 15 mg/kg of ConA and the livers were harvested 1 h later. The livers were soaked in ice-cold sterile Hank’s balanced salt solution supplemented with 0.5% fetal calf serum and subjected to two cycles of bead-beating using a TissueLyser (Qiagen, Valencia, CA, USA) followed by centrifugation. Bone marrow-derived neutrophil migration was determined using the TX ChemoTx^®^ System (101-3; NeuroProbe, Gaithersburg, MD, USA) consisting of a 3-μm-pore filter in a 96-well plate. Neutrophils were pre-labeled with 5 μM CellTracker Green (Molecular Probes, Eugene, OR, USA). Thirty microliters of liver lysates were placed in the wells and 20-μl droplets of 1.7 × 10^6^/ml pre-labeled neutrophils were placed in hydrophobic rings on the filter, which allowed contact with the liver lysates below. Additionally, a standard curve of stained cells was prepared from cell preparations, of which 30 μl was placed in separate wells and 25 μl RPMI 1640 supplemented with 5% FBS was added onto the filter top. The plate was incubated at 37 °C with 5% CO_2_ for 3 h. Following incubation, the medium in the top wells was absorbed, and any remaining cells were wiped off the top of the filter. The bottom plate (with the filter still on top) was centrifuged 430 × *g* for 5 min. The filter was removed and 100 nM phorbol myristate acetate (Sigma-Aldrich Chemical) was added to the wells and left for 20 min to facilitate permanent adhesion of transmigrated neutrophils; the cells were then washed with PBS. The plate was read on a Victor3 1420 Multilabel Counter (Perkin Elmer, Wellesley, MA, USA) at 485 nm excitation and 530 nm emission wavelengths. The numbers of migrated cells were calculated from the standard curves, based on relative fluorescence units.

### Isolation of primary mouse hepatocytes and *in vitro* TNFα treatment

Primary mouse hepatocytes were isolated from mice by perfusion with collagenase type I, as previously described^18^. The hepatocytes were cultured overnight, followed by treatment with recombinant TNFα (GIBCO BRL, Gaithersburg, MD, USA).

### Enzyme-linked immunosorbent assay for CXCL1 and CXCL2

The levels of CXCL1 or CXCL2 in the medium of TNFα-stimulated primary hepatocytes or liver lysates from ConA-treated mice were determined using the OptEIA mouse CXCL1 or CXCL2 enzyme-linked immunosorbent assay kit (ELISA) (BD Biosciences, San Diego, CA, USA) according to the manufacturer’s instructions.

### Vector constructs

Mouse *CXCL2* promoter (−900/+120) was PCR-amplified from mouse genomic DNA (Promega, Madison, WI, USA) and inserted into the pGL3 basic vector (Promega) using *Mlu*I and *Xho*I restriction enzyme sites. The pGL3-mCXCL2 AP1 mutant was generated by site-directed mutagenesis. All constructs were confirmed by DNA sequencing. pcDNA3-FLAG-SHP and pcDNA3-c-Jun were described previously^19^. Ad-GFP and Ad-FLAG-SHP were generated using the pAd-easy system (Clontech, Palo Alto, CA, USA). All viruses were purified using a CsCl gradient.

### Cell culture and transient transfection

HepG2 human hepatoma cells were maintained in Dulbecco’s modified Eagle’s medium (DMEM) supplemented with 10% FBS and antibiotics. AML12 (alpha mouse liver 12, a non-transformed mouse cell line) cells were cultured in DMEM/F-12 supplemented with insulin-transferrin-selenium, dexamethasone (40 ng/ml), and antibiotics. After overnight incubation in 5% CO_2_ at 37 °C, the medium was changed to serum-free culture medium. Vehicle control and TNF-α were added to the serum-free medium for the indicated times and at indicated concentrations. The cells were transiently transfected with Lipofectamine 2000 (Invitrogen, Gaithersburg, MD, USA) according to the manufacturer’s instructions. Total plasmid DNA for transfection was adjusted to 1 µg/well by adding an appropriate amount of empty vector, and pNL1.1. TK (Nluc/TK) plasmid (Promega) was used as an internal control. The cells were harvested 24–36 h post-transfection for firefly and Nano-Glo luciferase assays. The firefly luciferase activity was normalized to Nano-Glo luciferase activity.

### ChIP assay

The ChIP assay was performed according to the manufacturer’s instructions (EMD Millipore, Temecula, CA, USA). Briefly, AML12 cells were fixed with 1% formaldehyde and then harvested. Soluble chromatin was immunoprecipitated with an antibody against c-Jun (Cell Signaling Technology). After recovering of the DNA, PCR was carried out using primers encompassing the mouse *CXCL2* promoter (forward 5′-GAGGATTTGGGGAAGGACAT-3′, reverse 5′-CTCATCAGGAAGCACAGAGC-3′).

### Statistical analysis

Numerical data are presented as the mean ± SEM. All statistical analyses were performed using JMP software (SAS International Inc., Cary, NC). Student’s *t*-test was used for comparing two experimental conditions. Comparisons of multiple groups were performed using Tukey–Kramer HSD test after the one-way ANOVA. The survival ratio was analyzed using the log-rank test. The threshold of significance was set at *P * < 0.05.

## Results

### SHP deficiency in liver parenchymal cells is associated with increased susceptibility to ConA-induced hepatitis

**Figure 1:**
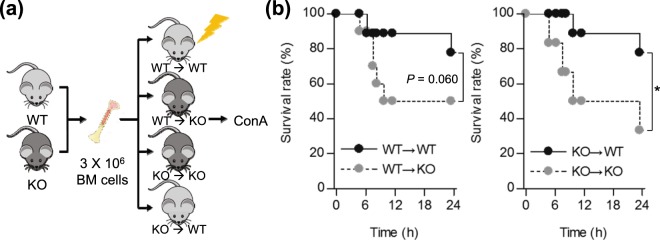
SHP deficiency in liver parenchymal cells increases mortality with lethal dose of ConA. (**a**) Schematic representation of the experimental set-up for preparing bone marrow chimeric mice; WT mice reconstituted with WT (WT → WT) or *Shp* KO (KO → WT) bone marrow-derived cells, and *Shp* KO mice reconstituted with *Shp* KO (KO → KO) or WT (WT → KO) bone marrow-derived cells. (**b**) Survival rate of mice injected with 27.5 mg/kg of ConA (n = 6–10 per group). Data are means ± SEMs. *P < 0.05 (log-rank test).

**Figure 2:**
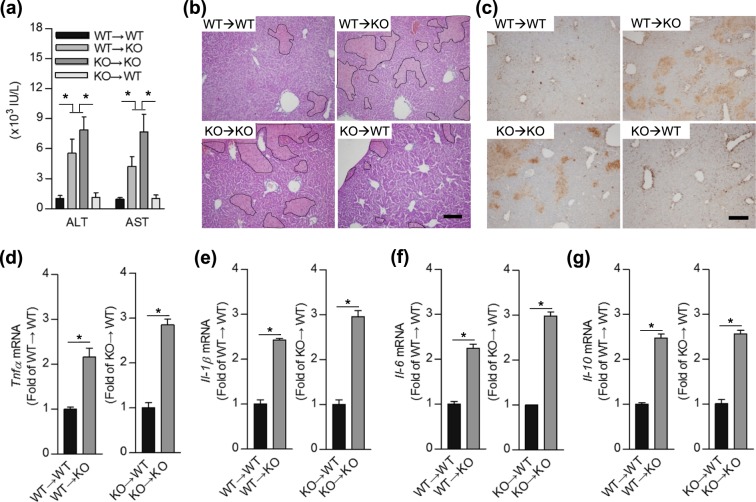
SHP deficiency in liver parenchymal cells is associated with increased liver damage during ConA-induced hepatitis. (**a**) Chimeric mice were challenged with 30 mg/kg of ConA, and plasma ALT and AST levels were measured after 9 h (n = 3–7 per group). (**b**-**g**) Chimeric mice livers were collected 24 h after 20 mg/kg of ConA injection (n = 3–8 per group). (**b** and **c**) Representative either (**b**) H&E or (**c**) TUNEL staining images are shown. Black dotted lines in highlight necrotic area and TUNEL-positive cells are colored brown. Scale bar, 200 μm. (**d**-**g**) The bar graph represents mRNA expression levels of *Tnfα*, *Il-1β*, *Il-6*, or *Il-10*. Relative gene expression levels were normalized against the expression of 18 S rRNA. Data are means ± SEMs. *P < 0.05 (Tukey-Kramer HSD test after the one-way ANOVA).

Previously, it has been reported that SHP is involved in neutrophil recruitment through the regulation of CXCL2 expression in the ConA-induced liver injury model^17^. Here, we investigated which cell type mediates the previously identified SHP effect. To this end, BM chimeras were established by BM transplantation, as shown in (Fig. 1a). KO-recipient groups (WT → KO or KO → KO) displayed a higher ConA-induced lethality than WT recipient groups (WT → WT/KO → WT), regardless of whether reconstitution was performed with WT or KO BM cells (Fig. 1b**)**. Accordingly, plasma ALT and AST levels were significantly higher 9 h after ConA injection in KO recipients, regardless of the donor genotype (Fig. 2a). Furthermore, H&E and TUNEL staining showed massively increased hepatocyte death in KO recipients compared with WT recipients 24 h after ConA challenge (Fig. 2b,c). We measured the levels of inflammatory cytokines in liver collected from chimeric mice at 24 h after ConA treatment (Fig. 2d–g). Consistent with above described findings, *Tnfα*, *Il-1β*, *Il-6*, and *Il-10* gene expressions in the liver were substantially increased in KO recipients compared with WT recipients. In contrast to the obvious detrimental effect of SHP ablation in liver parenchymal cells (KO recipients), we observed a modest contribution of SHP in BM cells during ConA-induced hepatitis. From these results, we conclude that SHP deficiency in liver parenchymal cells is critical for enhanced liver damage in the ConA-induced hepatitis mouse model.

### SHP deficiency in liver parenchymal cells augments neutrophil recruitment in ConA-induced hepatitis

**Figure 3:**
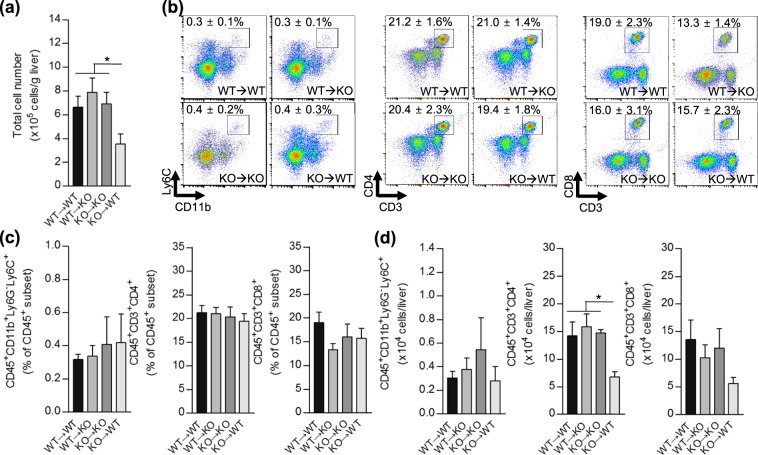
FACS analysis of infiltrating immune cells in ConA-treated chimeric mice liver. (**a**–**d**) Liver immune cells were isolated 3 h after challenge with 25 mg/kg of ConA and subjected to FACS analysis (n = 3–9 per group). For the identification of total isolated immune cell populations, cells were stained with fluorophore-conjugated different extracellular marker proteins: CD45, CD11b, CD3ε, Ly6G, Ly6C, CD4, or CD8a. (**a**) Absolute numbers of total immune cells are shown. (**b**) Representative FACS images are shown. (**c**,**d**) The bar graph represents **(c)** the ratio of each cell population to the total CD45^+^ subset (%) and (**d**) the absolute number of each type of immune cells. Data are means ± SEMs. *P < 0.05 (Tukey-Kramer HSD test after the one-way ANOVA).

**Figure 4:**
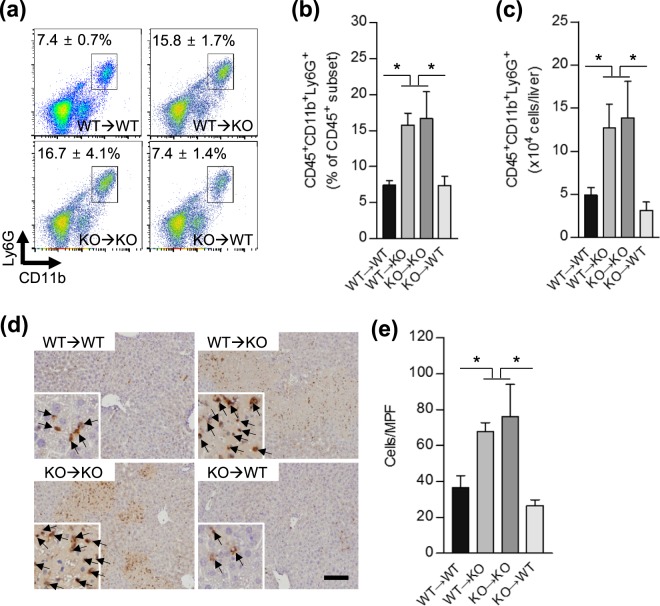
SHP deficiency in liver parenchymal cells promotes neutrophil infiltration to the injured liver. (**a**–**c**) Liver immune cells were isolated 3 h after challenge with 25 mg/kg of ConA and subjected to FACS analysis (n = 3–9 per group). For the identification of infiltrated neutrophil populations, cells were stained with fluorophore-conjugated different extracellular marker proteins: CD45, CD11b or Ly6G. (**a**) Representative FACS images are shown. (**b**,**c**) Bar graph represents (**b**) the ratio of the neutrophil population to the total CD45^+^ subset (%) and (**c**) the absolute numbers of neutrophils. (**d**,**e**) Immunohistochemistry and neutrophil count of liver samples 24 h after challenge with 20 mg/kg of ConA (n = 3–8 per group). Black arrows indicate neutrophils. Scale bar, 100 μm. MPF: medium power field. Data are means ± SEMs. *P < 0.05 (Tukey-Kramer HSD test after the one-way ANOVA).

To determine if parenchymal or non-parenchymal SHP affects immune cell recruitment to the injured liver in the ConA model, we purified infiltrating immune cells at 3 h after ConA challenge. FACS analyses revealed that numbers of infiltrating immune cells were significantly decreased in the liver samples of WT recipients that received BM from KO mice (KO → WT) (Fig. 3a). Additionally, absolute numbers of infiltrating T cells to the injured liver were significantly decreased in the KO → WT group. This decreased T cell population may influence the reduction in total cell numbers in this group. No significant monocyte/macrophage changes were observed among the different groups after ConA administration (Fig. 3b–d). Interestingly, neutrophil populations showed significantly different patterns based on the genotype of recipients. Notably, all KO-recipient groups displayed higher neutrophil infiltration than WT recipient groups. This increase in the ConA-induced neutrophil population was independent of the transplanted BM genotype (Fig. 4a–c). These findings were maintained 24 h after ConA treatment which were confirmed by immunohistochemical staining and quantification of neutrophils in liver (Fig. 4d,e). These data suggest that the extent of neutrophil migration to the injured liver and the severity of liver damage depend on the level of SHP in hepatic parenchymal cells.

### SHP affects neutrophil chemotaxis and controls TNFα-induced CXCL2, but not CXCL1 expression in mouse hepatocytes

**Figure 5:**
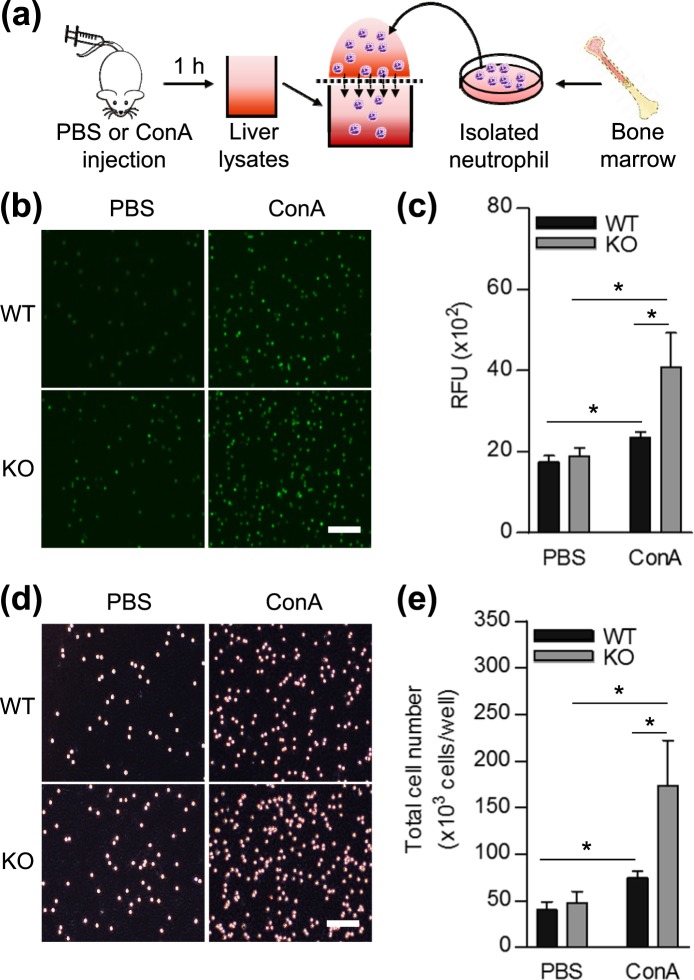
SHP deficiency in liver affects neutrophil migration *in vitro*. (**a**) *In vitro* neutrophil migration to the liver was determined using the TX ChemoTx System. Liver extracts were obtained from WT or *Shp* KO mice 1 h after either PBS or 15 mg/kg ConA-treatment (n = 3 per group), and neutrophils were freshly isolated from bone marrow of WT mice. Isolated neutrophils pre-labeled with CellTracker Green were placed in the upper chamber and liver extract was added to the lower chamber. (**b**,**d**) Representative images of migrated neutrophils are shown. (**b**) Fluorescence image and (**d**) bright field image. Scale bar, 100 μm. (**c**,**e**) Fluorescence density reads from the lower chamber were used to quantify neutrophil migration. RFU: relative fluorescence units. Data are means ± SEMs of at least 3 individual experiments. *P < 0.05 (Tukey-Kramer HSD test after the one-way ANOVA).

**Figure 6:**
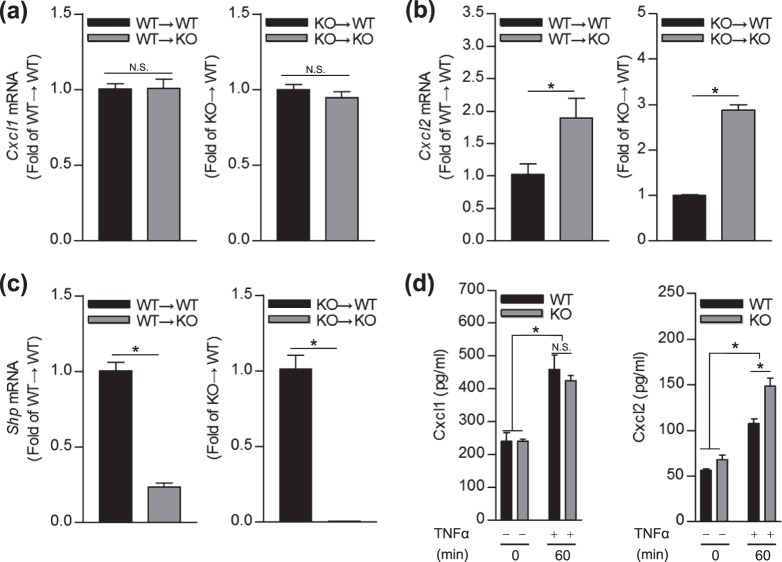
SHP differently regulates CXC-chemokine expression in mouse liver and primary hepatocytes. (**a**–**c**) Chimeric mice livers were collected 24 h after 20 mg/kg of ConA injection (n = 3–8 per group), and used for qPCR analysis of *Cxcl1*, *Cxcl2*, or *Shp* mRNAs. Relative gene expression levels were normalized against the expression of 18 S rRNA. (**d**,**e**) Primary hepatocytes isolated from WT and *Shp* KO mice were treated with recombinant mouse TNFα (30 ng/ml) for 60 min. (**d**) CXCL1 and (**e**) CXCL2 secretion in the culture supernatants were measured by ELISA in 3 individual experiments. Data are means ± SEMs. *P < 0.05 (Tukey-Kramer HSD test after the one-way ANOVA).

To evaluate whether SHP deficiency in the liver affects neutrophil migration, we conducted *in vitro* chemotaxis assays (Fig. 5a). Neutrophil migration toward liver lysates from ConA treated-*Shp* KO mice was higher than toward liver lysates from WT mice (Fig. 5b–e), indicating that intrinsic factors in the *Shp* KO mouse liver promote neutrophil migration in ConA-induced hepatitis. Recent reports have suggested that neutrophil migration to the injured liver is critically dependent on a hepatic chemokine network^20^. Therefore, we hypothesized that increased neutrophil migration toward the liver lysates of ConA-treated *Shp* KO mice may be influenced by C-X-C chemokines, which are major chemoattractants for neutrophils in various inflammatory and autoimmune diseases^21,22^. To test this hypothesis, we measured CXCL2 secretion first in liver lysates isolated from WT and *Shp* KO mice 1 h after ConA administration. Upon ConA stimulation, *Shp*-deficient liver was able to produce high level of CXCL2 than that of WT liver lysates (Supplementary Fig. 3). Next, we confirmed the levels of *Cxcl1* and *Cxcl2* expression in WT and *Shp* KO recipient mice liver following 24 h after ConA treatment. *Cxcl1* expression showed similar pattern among groups, but *Cxcl2* expression were significantly higher in KO recipients, regardless of the donor genotype (Fig. 6a,b). Under these situation, *Shp* expression was significantly suppressed in KO recipient liver, indicating that *Shp* is inversely correlated with *Cxcl2* expression (Fig. 6c). More specifically, we isolated primary hepatocytes, representative of hepatic parenchymal cells, from WT and *Shp* KO mice livers. It is well established that TNFα is a major proinflammatory cytokine involved in early inflammatory events and that TNFα stimulates CXCL2 expression in ConA-induced hepatitis^23^. We measured C-X-C chemokine levels after TNFα treatment in primary hepatocytes isolated from WT and *Shp* KO mice (Fig. 6d). CXCL1 secretion was significantly increased by TNFα stimulation; however, there were no significant differences between both groups. In contrast, CXCL2 secretion was largely up-regulated in SHP-deficient hepatocytes 60 min after TNFα treatment. Finally, we checked TNFα-induced CXCL2 secretion in primary hepatocytes isolated from *Shp* KO mice under the condition of SHP overexpression by adding Ad-SHP (Supplementary Fig. 4). TNFα-evoked CXCL2 secretion was significantly suppressed by SHP overexpression. These data indicate that hepatocytes are a major source for increased CXCL2 production and secretion in *Shp* KO mice, and SHP acts as a negative downstream regulator of TNFα-mediated CXCL2 in hepatocytes.

### SHP regulates CXCL2 transcription through interaction with c-Jun

**Figure 7:**
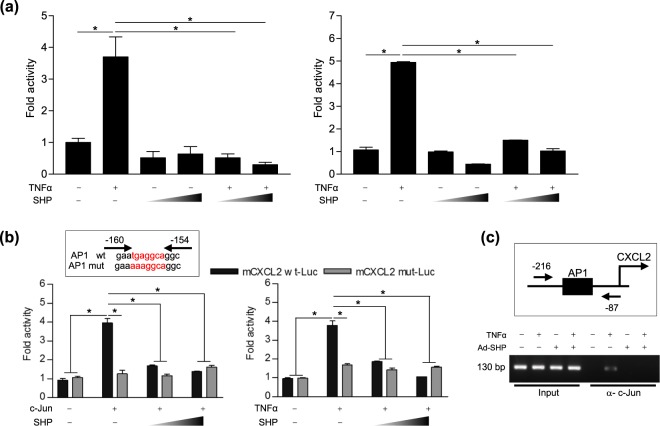
SHP negatively controls CXCL2 transcription through the regulation of binding between c-Jun and the AP-1 site. (**a**) AML12 and HepG2 cells were transfected with vector expressing the mouse CXCL2 (mCXCL2) promoter (200 ng per well) and then treated with TNFα (30 ng/ml) with or without co-transfection with SHP. (**b**) AML12 cells were transfected with vector expressing AP-1 WT or AP-1 mutant mCXCL2 promoter (200 ng per well). The cells were co-transfected with the indicated plasmids and/or treated with TNFα (30 ng/ml). Firefly luciferase activity was normalized to Nano-Glo luciferase activity. (**c**) AML12 cells were transfected with vector expressing mCXCL2 promoter and then treated with TNFα (30 ng/ml) in the presence of Ad-GFP or Ad-SHP. Input represents 10% of purified DNA in each sample. Soluble chromatin was immunoprecipitated with antibody against c-Jun and purified DNA samples were analyzed by PCR with primers encompassing the AP-1 site of the *mCxcl2* gene promoter. Data are means ± SEMs of 3 individual experiments. *P < 0.05 (Tukey-Kramer HSD test after the one-way ANOVA).

Finally, we investigated the mechanism of *Cxcl2* expression by SHP. Firstly, we tested whether SHP affects TNFα-induced *Cxcl2* promoter activity through a reporter luciferase assay in AML12 and HepG2 cells. In both cell lines, overexpression of SHP drastically suppressed TNFα-induced *Cxcl2* promoter activity (Fig. 7a). A previous study reported that *Cxcl2* is regulated by NF-κB and c-Jun/Activator Protein-1 (AP-1). In our previous study, we showed that the effect of SHP on *Cxcl2* transcription is not influenced by the NF-κB pathway^17^. Therefore, we used reporter assays with AP-1-mutant *Cxcl2* promoter constructs to examine the participation of the AP-1 site in the SHP regulatory effect. Our data show that c-Jun/TNFα-mediated WT but not mutant *Cxcl2* promoter activity was remarkably suppressed by SHP overexpression (Fig. 7b). In addition, ChIP assays showed that TNFα-induced binding of c-Jun to the AP-1 site of the *Cxcl2* promoter is almost entirely abrogated by the overexpression of SHP (Fig. 7c). Taken together, these results demonstrate that the inhibitory effect of SHP on TNFα-mediated CXCL2 expression is mainly through the inhibition of c-Jun–DNA binding.

## Discussion

Previously, we observed that *Shp* KO mice show higher neutrophil trafficking to the injured liver following ConA treatment, accompanied by enhanced CXCL2 expression^17^. Here, using bone marrow chimeric mice, we verified that SHP is a previously unrecognized transcriptional regulator of hepatic CXCL2 that contributes to the pathogenesis of fulminant hepatitis. *Shp* KO recipient mice, regardless of the donor genotype, were highly susceptible to ConA-induced liver injury and prone to death upon ConA administration. Infiltrating neutrophils to the injured liver, driven by CXCL2, were also significantly increased in KO recipient mice regardless of WT or *Shp* KO donor, suggesting that SHP deficiency in liver parenchymal cells is critical for ConA-induced hepatitis. Intriguingly, SHP in hepatocytes differentially regulates CXCL1 and CXCL2, and negatively controls TNFα-mediated CXCL2 expression through inhibition of c-Jun-DNA binding.

In ConA-induced hepatitis, TNFα and IFNγ are rapidly produced in response to ConA and induce the expression of pro-inflammatory proteins in both parenchymal and non-parenchymal liver cells^24^. Several studies have reported that CXCL2 is secreted by various hepatic cells under different pathological conditions^25,26^, and mouse CXCL2 is mostly induced by TNFα. Moreover, CXCL2 production stimulated by TNFα partially contributes to liver injury mediated through neutrophil recruitment^23^. We observed that SHP deficiency in hepatocytes led to the increased secretion of CXCL2, but not CXCL1, upon TNFα stimulation, emphasizing that hepatocytes contribute to increased levels of circulating CXCL2 in *Shp* KO mice during ConA-induced hepatitis. This is consistent with our previous study that demonstrates increased CXCL2 expression, but not CXCL1, in both the liver and plasma of *Shp* KO mice after ConA administration. Moreover, it has been demonstrated that *Shp* expression was inversely correlated with *Cxcl2* expression in ConA-treated livers^17^, implying that SHP is a possible transcriptional regulator of CXCL2.

Here, to solve the remaining question whether *Shp* KO neutrophils have an enhanced ability to chemotactically migrate to the injured liver, we confirmed chemokine receptor CXCR2 expression on cell membrane of circulating neutrophils as well as other immune cells after ConA treatment using FACS (Supplementary Fig. 5). The CXCR2 is expressed at high levels on cell membrane of circulating neutrophils, and is critical for directing their migration to sites of inflammation^27^. In this study, basal levels of CXCR2 membrane expression on neutrophils were not different between WT and *Shp* KO mice. Activation of Toll-like receptors (TLRs) in neutrophil downregulates CXCR2 membrane expression, and impairs neutrophil migration^28^. It has been reported that ConA induces expression of TLRs in murine macrophages, and renders them more susceptible to subsequent activation by TLR ligands^29^. In these regards, it might be assumed that ConA treatment could regulate CXCR2 expression on cell membrane through modulation of TLR signalling. In the present study, ConA administration lowered CXCR2 membrane expression on neutrophils, which would be regulated by ConA treatment-mediated regulation of TLR signalling. However, these decreased CXCR2 membrane expressions in neutrophil were comparable between WT and *Shp* KO mice. In regarding to monocytes, CD8^+^ T cells, and CD4^+^ T cells, CXCR2 membrane expressions were similar between WT and *Shp* KO mice, and not influenced by ConA treatment. These results demonstrated that hepatic CXCL2 is associated with augmented neutrophil recruitment to the injured liver of *Shp* KO mice in a neutrophil CXCR2 expression modulation independent manner.

Furthermore, we addressed the question of why CXCL1 and CXCL2 are differentially regulated in the absence of SHP during the ConA-induced inflammatory response. *Cxcl1* and *Cxcl2* share an NF-κB-binding site in their promoter regions^30^. Intriguingly, NF-κB inhibition effectively prevented the up-regulation of *Cxcl1* but not *Cxcl2*. Complete suppression of *Cxcl2* required the dual inhibition of NF-κB and AP-1^31^. Here, we report that SHP specifically controls the transcription of *Cxcl2*, but not *Cxcl1*, by directly inhibiting the binding of c-Jun to the AP-1 site within the *Cxcl2* promoter. We targeted the AP-1 site to exclude the possibility of other transcription factors sharing a promoter region with *Cxcl1*. Additionally, human and mouse *Cxcl2* share NF-κB- and AP-1-binding sites in their promoter regions^32^, suggesting evolutionary conservation of the identified mechanisms. Hence, we propose that the targeting of SHP might represent a novel therapeutic option in various human inflammatory diseases, including hepatitis.

SHP has been previously reported to negatively regulate Toll-like receptor-induced inflammatory responses in innate immune cells through a biphasic interaction with its cytoplasmic partners TRAF6 and NF-κB p65^15^. We now found that SHP deficiency in liver parenchymal cells aggravates neutrophil-mediated liver injury via modulation of CXCL2 expression in an NF-κB independent manner, suggesting that SHP may have cell type-specific roles in the control of inflammatory processes.

Collectively, we demonstrated that CXCL2-derived neutrophils are critical mediators of experimental hepatitis. We have revealed a novel function of SHP as a negative transcriptional regulator of hepatocyte-derived CXCL2. Therefore, the modulation of SHP may have therapeutic benefits in the treatment of inflammatory liver diseases.

## Electronic supplementary material

Supplementary information
