# Supplementary material for: Small heterodimer partner negatively regulates C-X-C motif chemokine ligand 2 in hepatocytes during liver inflammation
Source: Sci Rep. 2018 Oct 15;8:15222. doi: 10.1038/s41598-018-33660-z (PMC6189097; doi:10.1038/s41598-018-33660-z)
Supplement: Supplementary file 1 — Supplementary information [file 41598_2018_33660_MOESM1_ESM.pdf]

Small heterodimer partner negatively regulates C-X-C motif chemokine ligand 2 in hepatocytes during liver inflammation

Running title: The role of SHP in hepatic CXCL2 regulation

Jung-Ran Noh, Yong-Hoon Kim, Don-Kyu Kim, Jung Hwan Hwang, Kyoung-Shim Kim, Dong-Hee Choi, Seon-Jin Lee, Hee Gu Lee, Tae Geol Lee, Hong-Lei Weng, Steven Dooley, Hueng-Sik Choi* & Chul-Ho Lee*

Supplementary figure 1.

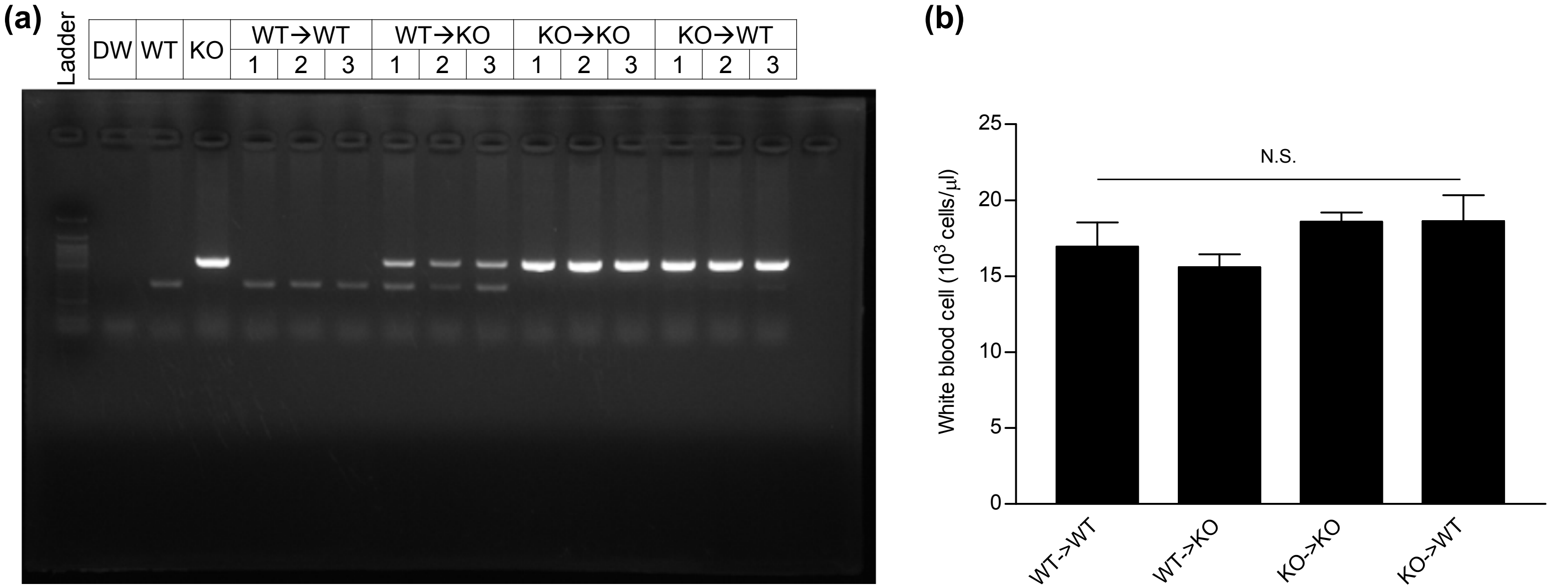

Supplementary Figure 1. *Shp* genotype analysis and white blood cell count in bone-marrow chimeras.

Bone marrow chimeras were produced by bone marrow transplantation (n=8-9 per group). At 8 weeks after transplantation, genomic DNA was extracted from peripheral blood of the chimeric mice and used for (a) genotype analysis of the *Shp* gene and (b) white blood cell counts. Data are means ± SEMs; WT mice reconstituted with WT bone marrow-derived cells (WT→WT); *Shp* KO mice reconstituted with WT bone marrow-derived cells (WT→KO); *Shp* KO mice reconstituted with *Shp* KO bone marrow-derived cells (KO→KO); WT mice reconstituted with *Shp* KO bone marrow-derived cells (KO→WT). Data are means ± SEMs. N.S. = no significant difference (Tukey-Kramer HSD test after the one-way ANOVA).

Supplementary figure 2.

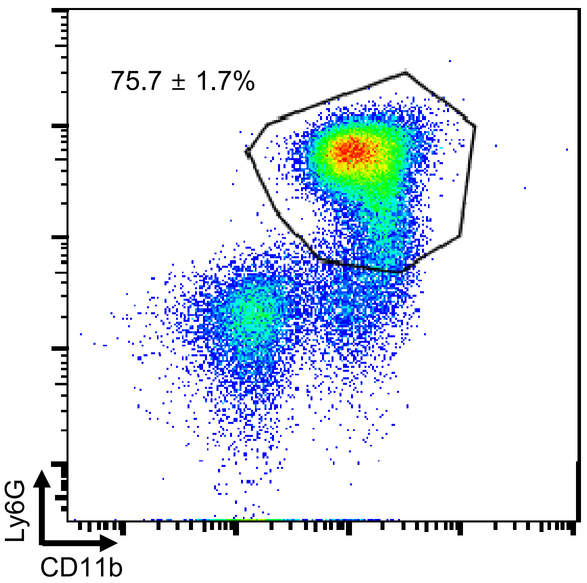

Supplementary Figure 2. Purity assessment of bone marrow derived neutrophils by FACS analysis. Mice were euthanized and the bone marrow was collected from the femur, tibia, pelvis, scapula, and humerus. Bone marrow neutrophils were isolated using a Percoll density gradient. For the identification for isolated neutrophils, cells were stained with fluorophore-conjugated antibodies against extracellular marker proteins CD11b and Ly6G. Representative FACS images are shown. Double-positive cells were considered neutrophils. Neutrophil preparations were routinely >75% pure.

Supplementary figure 3.

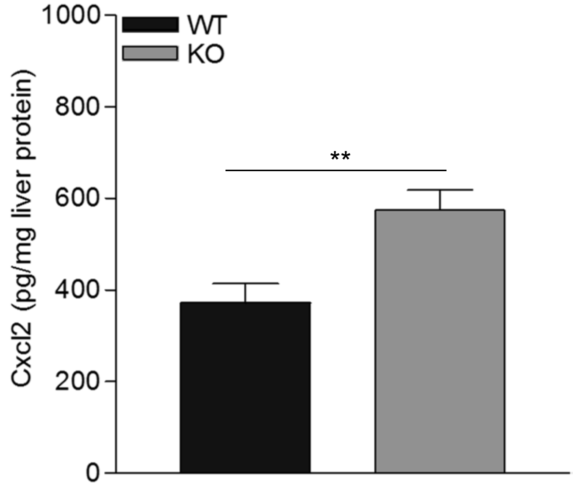

Supplementary Figure 3. CXCL2 protein level increases in SHP deficiency. WT and Shp KO mice were treated with 15 mg/kg of ConA (n=6-7) and the livers were harvested 1 h after. CXCL2 protein levels in the liver lysates were measured by ELISA. Data are means ± SEMs. ***P* < 0.01 (Student's *t*-test).

Supplementary figure 4.

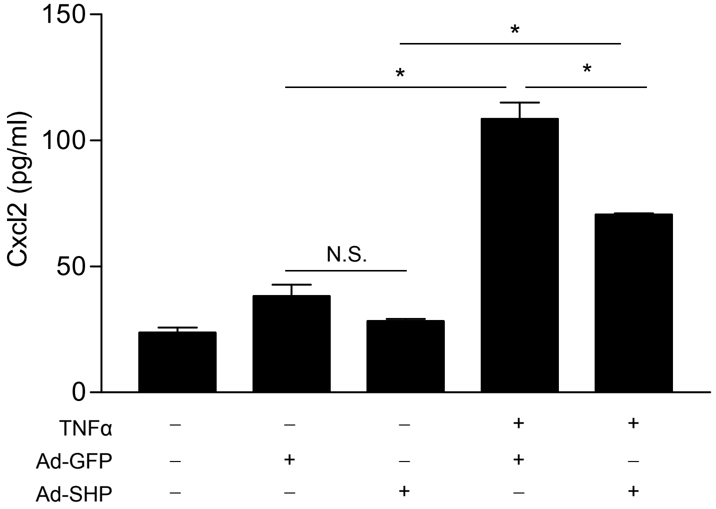

Supplementary Figure 4. SHP overexpression inhibits TNFα-evoked increase of CXCL2 secretion in SHP-deficient mouse primary hepatocytes. Primary hepatocytes isolated from *Shp* KO mice were treated with TNFα (30 ng/ml) for 1 h in the presence of Ad-GFP or Ad-SHP. CXCL2 secretion in the culture supernatants were measured by ELISA. Data are means ± SEMs of at least 3 individual experiments. *$P < 0.05$ and N.S. = no significant difference (Tukey-Kramer HSD test after the one-way ANOVA).

Supplementary figure 5.

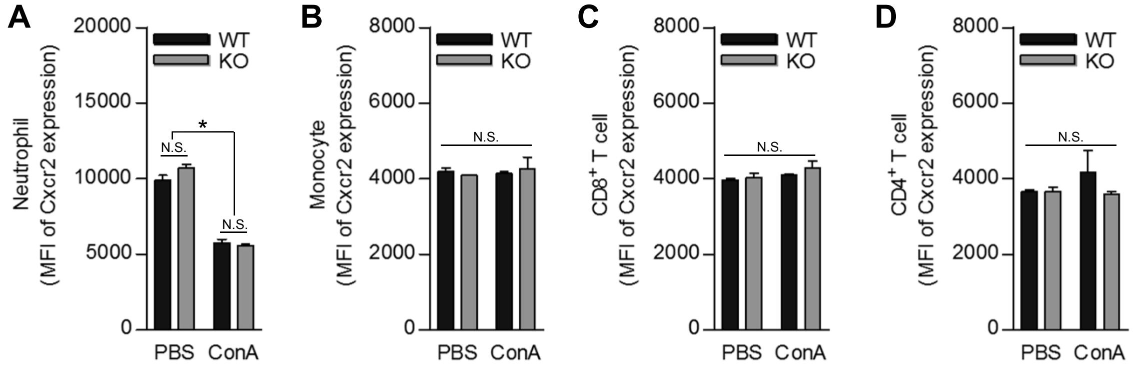

Supplementary Figure 5. Assessment of cell surface Cxcr2 expression by FACS analysis. Peripheral blood cells were freshly collected from mice 3 h after challenge with 25 mg/kg ConA (n=3 per group). Blood neutrophils, monocytes, $CD8^+$ T cells and $CD4^+$ T cells were analyzed for cell surface CXCR2 expression by FACS. Cells were stained with fluorophore-conjugated different extracellular marker porteins: CD45, CD11b, CD3ε, Ly6G, Ly6C, CD4, CD8a, or CXCR2. Graphs show mean fluorescence intensity (MFI) of CXCR2. Data are means ± SEMs. $*P < 0.05$ and N.S. = no significant difference (Tukey-Kramer HSD test after the one-way ANOVA).

Supplementary figure 6.

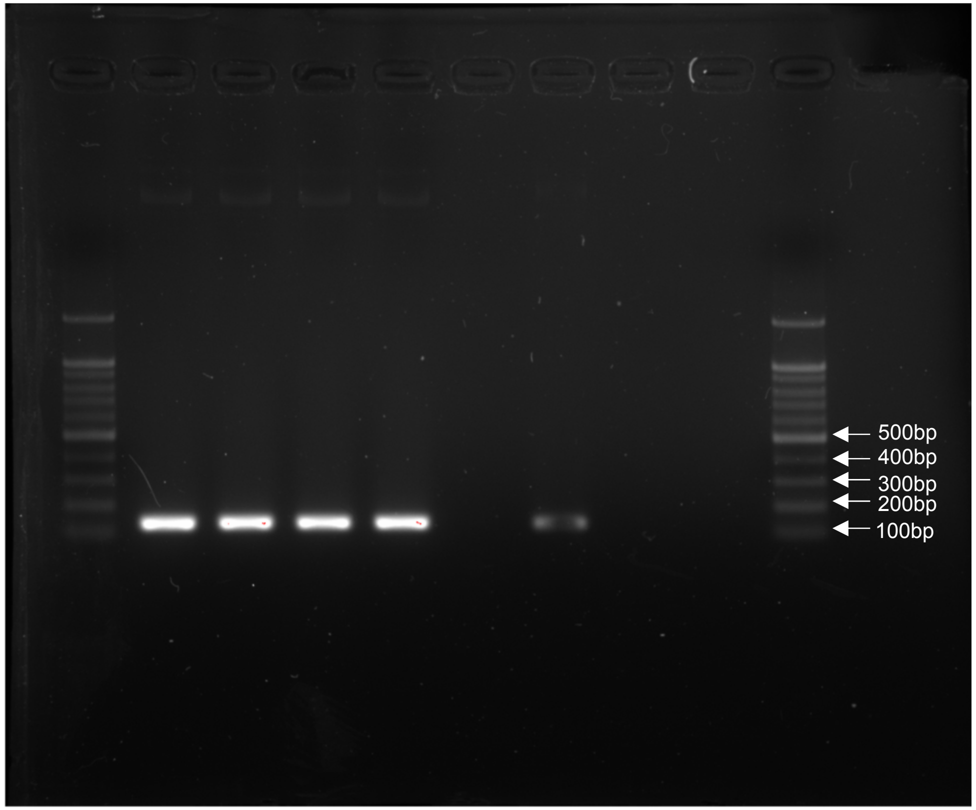

Supplementary Figure 6. Full-length gel image for Figure 7c.

Supplementary table 1. Sequences of PCR primers used in this study

Gene	Gene Bank Accession Number	Primer Sequence
Tnfα	NM_013693.3	(F) 5’- TGGCCTCCCTCTCATCAGTT -3’<br>(R) 5’- CCTCCACTTGGTGGTTTGCT -3’
Il-1β	NM_008361.4	(F) 5’- CTACAGGCTCCGAGATGAACAAC -3’<br>(R) 5’- TCCATTGAGGTGGAGAGCTTTC -3’
Il-6	NM_031168.2	(F) 5’- TTCCATCCAGTTGCCTTCTTG -3’<br>(R) 5’- GGGAGTGGTATCCTCTGTGAAGTC -3’
Il-10	NM_010548.2	(F) 5’- GGGTTGCCAAGCCTTATCG -3’<br>(R) 5’- TCTCACCCAGGGAATTCAAATG -3’
Cxcl1	NM_008176.3	(F) 5’- TGTCAGTGCCTGCAGACCAT -3’<br>(R) 5’- CAAGGGAGCTTCAGGGTCAA -3’
Cxcl2	NM_009140.2	(F) 5’- GGCTGTTGTGGCCAGTGAA -3’<br>(R) 5’- CGCCCTTGAGAGTGGCTATG -3’
Shp	NM_011850.3	(F) 5’-TCTGCAGGTCGTCCGACTATT-3’<br>(R) 5’-TGTCTTGGCTAGGACATCCA-3’
18s rRNA	NR_003278.3	(F) 5’- GACACGGACAGGATTGACAGATTGATAG -3’<br>(R) 5’- GTTAGCATGCCAGAGTCTCGTTCGTT -3’
